# Coordinative Combination of Nitroamine and Gem-Dinitromethyl with Fused Ring for Enhanced Oxygen Balances and Detonation Properties

**DOI:** 10.3390/ijms232214337

**Published:** 2022-11-18

**Authors:** Xun Zhang, Yaxi Wang, Xinyuan Zhao, Chunlin He, Siping Pang

**Affiliations:** 1 School of Materials Science & Engineering, Beijing Institute of Technology, Beijing 100081, China; 2 Experimental Center of Advanced Materials, School of Materials Science & Engineering, Beijing Institute of Technology, Beijing 100081, China; 3 Yangtze Delta Region Academy, Beijing Institute of Technology, Jiaxing 314019, China; 4 Chongqing Innovation Center, Beijing Institute of Technology, Chongqing 401120, China

**Keywords:** nitroamine, gem-dinitromethyl, high nitrogen-oxygen content, triazolo-tetrazine fused ring

## Abstract

Oxygen balance and heat of formation are closely related to the nitrogen and oxygen content in a molecule and have a significant effect on the detonation performance of energetic materials. Here a new family of 1,2,4-triazolo [4,3-b][1,2,4,5]-tetrazine containing gem-dinitromethyl and nitroamine with high nitrogen-oxygen content was synthesized and characterized. Moreover, the structure of the guanidine salt (**3**) and TATOT salt (**4**) were confirmed by single-crystal X-ray diffraction. The nitrogen and oxygen content of ammonium salt **2** reached 82.5%, with a high density (1.805 g cm^−3^) and high detonation properties (*D* = 8900 m s^−1^; *P* = 32.4 GPa), which were similar to those of RDX.

## 1. Introduction

**Figure 1:**
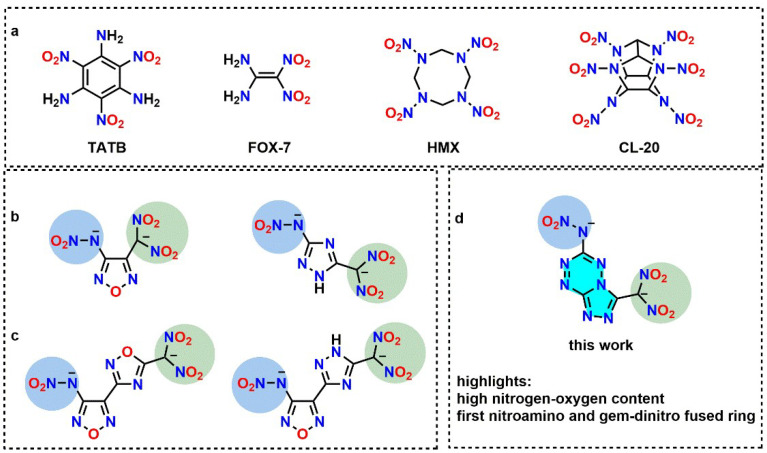
(**a**) Structures of TATB, FOX-7, HMX and CL-20. (**b**) Gem-dinitromethyl (green circle) and nitroamine groups (blue circle) substituted on a single ring. (**c**) Gem-dinitromethyl and nitroamine groups substituted on a linked ring. (**d**) Gem-dinitromethyl and nitroamine groups substituted on a fused ring (bright blue area).

The design and synthesis of energetic compounds with both enhanced energy and safety are highly desired in the field of energetic materials. Due to the existence of the energy and safety contradiction, higher energy usually leads to lower safety. The energetic compounds involving a single explosophoric group always result in only one of the energy or safety properties being improved. For instance, although increased detonation performances were obtained for the nitroamine benchmark explosives such as 1,3,5,7-tetranitro-1,3,5,7-tetrazoctane (HMX) or hexanitrohexaazaisowurtzitane (CL-20), the large number of nitroamine groups in the structure resulted in a decreased safety with sensitivities located in the sensitive range (Figure 1a). The coordinative combination of different explosophoric groups is beneficial for achieving enhanced comprehensive properties for energetic compounds; the representative examples, such as 2,4,6-triamino-1,3,5-trinitrobenzene (TATB) and 1,1-diamino-2,2-dinitroethene (FOX-7), are insensitive explosives due to the existence of C-NH_2_ and C-NO_2_ groups [1,2,3]. Demonstrating the coordinative combination of an explosophoric group is an effective way to tune the structures of energetic compounds to gain desired properties [4,5].

Due to the limited number of explosophoric groups, the construction of novel backbones is a commonly used strategy for designing new energetic compounds. The nitrogen-rich conjugated coplanar system has the advantages of high heat of formation (HOF) and ring-strain energy to store more chemical energy, and has therefore become a considerable choice as the skeleton for high energy density materials (HEDMs) [6]. Lots of fused heterocyclic ring-based energetic compounds preserving good thermal stability and low sensitivities have been designed and synthesized [7]. A higher chemical energy storage for the skeleton which is reflected in forms of higher heat of formation usually consists of more nitrogen atoms in the conjugated system. However, the increase of the nitrogen atoms in the skeleton always results in a decreased reactivity and makes it difficult to introduce nitro groups directly bonded to the fused ring. To maintain the maximum nitrogen atoms as well as two substituted positions in the structure, the 1,2,4-triazolo[4,3-b][1,2,4,5]tetrazine fused backbone, which can be readily constructed by reacting hydazinyl-1,2,4,5-tetrazines with cyanogen bromide, is an ideal option for designing novel energetic compounds.

The combination of gem-dinitromethyl [8] and nitroamine [9] groups with bridged heterocycles or single five member ring such as furazan-1,2,4-triazole, furazan-1,2,4-oxadiazole, 1,2,4 triazole or furazan were previously reported to access novel energetic compounds (Figure 1b,c) [10,11,12,13,14]. Most of those compounds exhibit improved detonation properties and oxygen balances; however, a high sense of unsatisfactory sensitivity was also observed, which limits their practical applications [15,16]. Incorporating a suitable backbone with gem-dinitromethyl and nitroamine groups to gain enhanced stabilities would be a good promotion for further applications. Triazole–tetrazine fused backbone has found utility in designing new HEDMs with enhanced detonation properties and stability for its large conjugated system [17,18]. In this work, a series of triazole–tetrazine energetic salts **1**–**4** containing gem-dinitromethyl and nitroamine energetic groups have been successfully synthesized (Figure 1d). Their properties were tuned by incorporation with different cations. The structures of the new compounds were characterized by multinuclear NMR, elemental analyses, IR and X-ray single crystal diffraction. It is interesting to note that all the prepared salts show good sensitivity to mechanical stimuli, which is a promising perspective for practical applications.

## 2. Results and Discussion

### 2.1. Synthesis

**Scheme 1:**
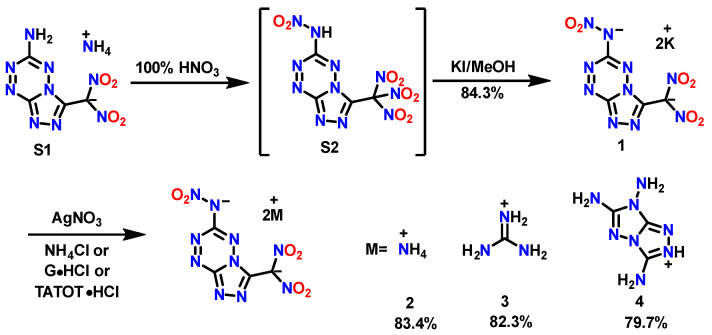
Synthesis of dipotassium salt **1**, diammonium salt **2**, diguanidine salt **3** and di-TATOT salt **4**.

The synthetic route is shown in Scheme 1. The starting material, compound **S1**, was obtained using a special aminohydrazone cyclization strategy based on our previous research [18]. Then, the further nitration of **S1** in 100% nitric acid creates the fused triazole–tetrazine nitroform compound **S2**. Pure **S2** can be isolated by column chromatography; **S2** is stable in solvent but will decompose fast in a solid state. The dipotassium salt **1** was readily prepared in good yield by reacting **S2** with potassium iodide in methanol. However, if the potassium iodide was replaced by hydroxylamine hydrochloride or potassium hydroxide, the reduction of nitroform proceeded incompletely due to the weak reducibility of hydroxylamine. The attempt to prepare the hydrazinium salt by reacting diluted hydrazine hydrate, which has a strong reducing ability, resulted in a significant decomposition of **S2**. Subsequently, the diammonium salt **2** (yield: 83.2%), diguanidine salt **3** (yield: 82.1%) and di-TATOT salt **4** (yield: 79.5%) were prepared through a metathesis reaction using silver salts with corresponding chloride salt.

### 2.2. Single-Crystal X-ray Analysis

**Figure 2:**
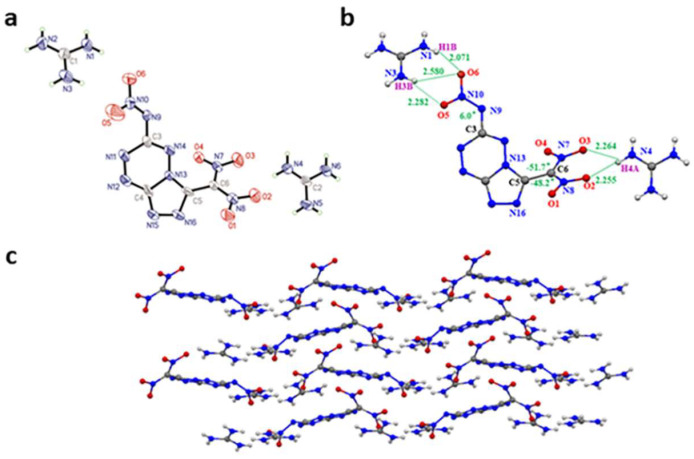
(**a**) Molecular structure of **3**. (**b**) Hydrogen bonds of **3** (green dash line). (**c**) Crystal packing diagram of **3**.(blue for nitrogen atoms and red for oxygen atom).

Crystals of (3-(dinitromethanidyl)-[1,2,4]triazolo[4,3-b][1,2,4,5]tetrazin-6-yl)(nitro)amide guanidine salt (**3**) (CCDC 2204894) were obtained from water. Compound **3** belongs to the monoclinic crystal system with space group *Cc* (Z = 4) symmetry with a crystal density of 1.629 g cm^−3^ (296 K). The X-ray structure and crystal parameters of compound **3** are shown in Figure 2a and Table S1. From Figure 2b, the torsion angle N3-C9-C10-O5 = 6° shows acceptable planarity of the nitramine with the fused ring. The dinitromethyl group and the fused heterocyclic ring form the torsion angle N13-C5-C6-N7 = −51.7° and N16-C5-C6-N8 = −48.2°. Also, the guanidine cations form a large number of intramolecular O⋯H hydrogen bonds with the geminal dinitro and nitroamine groups. The distances of O⋯H are in the range of 2.071–2.580 Å (Figure 2b). A mixed stacking was observed in the packing diagram of **3** (Figure 2c).

**Figure 3:**
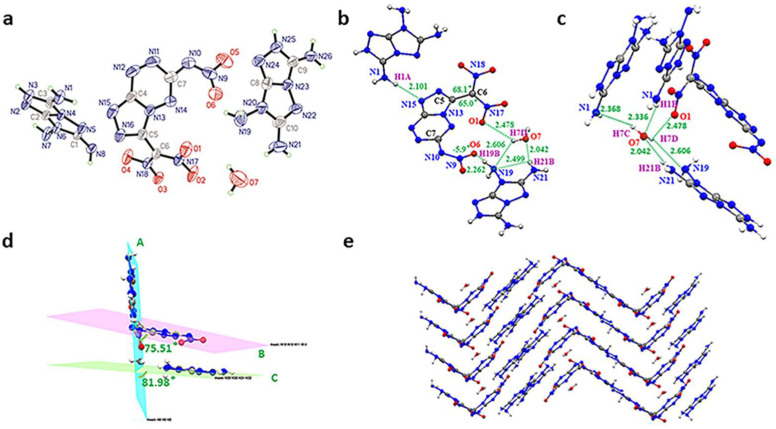
(**a**) Molecular structure of **4**. (**b**) Hydrogen bonds of **4** (green dash line). (**c**) Hydrogen bonds (green dash line). resulting with H_2_O to connect molecules to form layers. (**d**) Planarity of **4** (bright blue for plane A, purple for plane B, green for plane C). (**e**) Crystal packing diagram of **4** (blue for nitrogen atoms, red for oxygen atoms).

Crystal of the TATOT salt (**4**·H_2_O) (CCDC 2204893) was grown from methanol with a crystal density of 1.760 g cm^−3^ (298K) (Figure 3a). Compound **4** crystallizes in the monoclinic crystal system and belongs to the P2_1_-c (Z = 4) symmetry. Similar to guanidine salt, the geminal dinitro group is twisted totally out of the plane with N18–C6–C5–N16 = 68.1° and N17–C6–C5–N13 = 65.0° (Figure 3b). As shown in Figure 3d, the dihedral angles of the fused ring plane B and another TATOT cation plane C with plane A are 75.51° and 81.98°, respectively, implying that the fused ring is substantially parallel to plane C. Meanwhile, the asymmetric unit of **4** contains one water molecule as a significant link by forming hydrogen bonds (HBs) interaction. First, the water forms three intramolecular HBs in the same layer. Then, the O7-H7C…N1 and N1-H1B…O7 HBs connect the water with another two molecules in a different direction (Figure 3c). Thus, abundant hydrogen bonds between compound **4** and H_2_O molecule aid in wave-like packing (Figure 3e).

### 2.3. Sensitivity

Sensitivity is an important physical index for the secure use of energetic materials. The sensitivity was evaluated by using standard BAM testers. As shown in Table 1, all the new synthesized compounds, including metal salt, have lower sensitivity. The guanidine salt (**3**) possesses an impact sensitivity of 40 J, and friction sensitivity of 360 N, which can be attributed to the strong hydrogen bond network. Due to the slip action of the wave-like stacking, the TATOT salt (**4**) shows a lower impact sensitivity (40 J) and friction sensitivity (160 N), indicating the contributions to reduced sensitivity.

**Figure 4:**
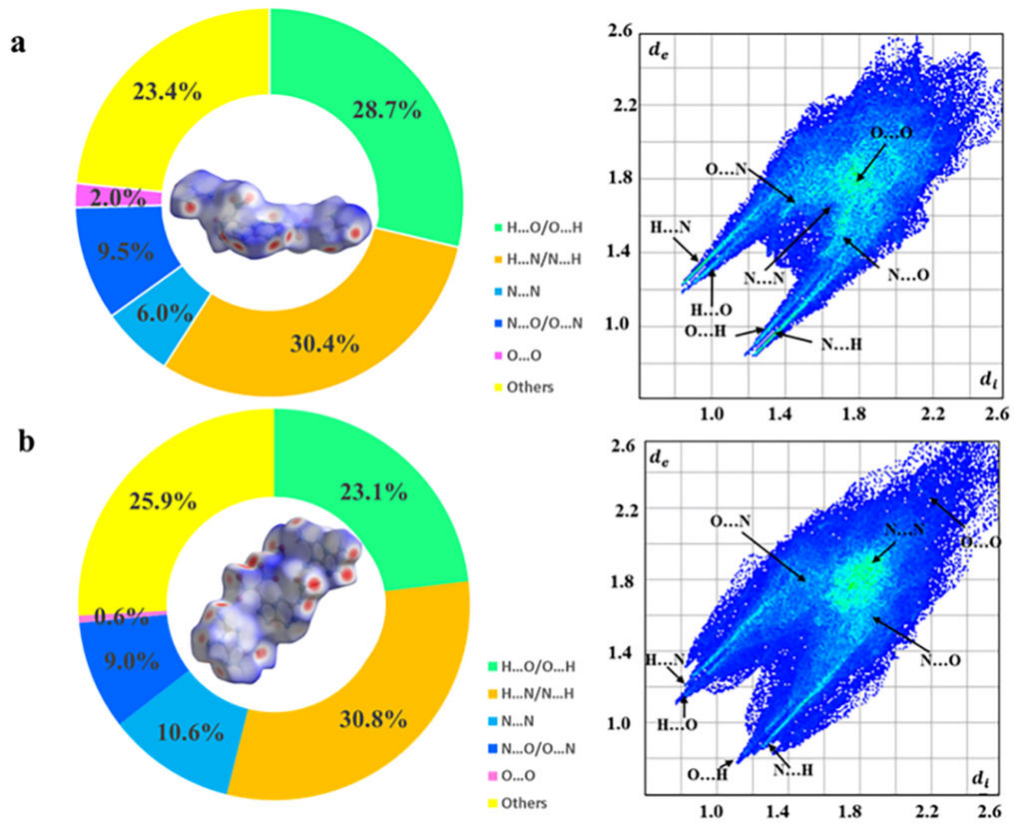
Hirshfeld surface analyses for **3** (**a**) and **4** (**b**).

To better understand the reasons for low sensitivity, Hirshfeld surfaces and the associated two-dimensional fingerprint plots were introduced using the software CrystalExplorer 17.5 as shown in Figure 4a,b [19,20]. The red spots on the Hirshfeld surface represent high close contact areas. The red dots are mainly concentrated on the sides of the surface, representing the close HBs interactions between O…H and N…H. The percentage contribution of each type of interaction is aggregated according to the 2D fingerprint. O…H and N…H interactions in salts **3** and **4** play a major role, accounting for 59.1% and 53.9% of the total weak interactions, respectively, indicating the advantage of the existence of extensive hydrogen bonding interactions for improving molecular stability. These abundant hydrogen bonds also help to improve molecular thermal stability for the new energetic salts. The onset decomposition temperatures of salt **3** (228.4 °C) are comparable to that of RDX (204 °C), as shown in Figure S15 and Table 1.

### 2.4. Physicochemical and Energetic Properties

The heat of formation (HOF) for these new energetic salts was calculated by the Gaussian 09 (Revision E.01) suite of programs using isodesmic reactions (Scheme S1, Supplementary Materials). The potassium salt **1** (172.6 kJ mol^−1^) exhibits positive HOFs based on the extensive N-N bonds of this new triazole–tetrazine anion, higher than the traditional explosive RDX (70.3 kJ mol^−1^). Meanwhile, TATOT salt **4** has the highest heat of formation of 1627.3 kJ mol^−1^, followed by guanidine salt **3** (485.1 kJ mol^−1^) and ammonium salt **2** (439.7 kJ mol^−1^). The detonation velocity (*D*) and detonation pressure (*P*) of these new compounds compared with traditional explosives RDX were summarized based on the density, which were measured by using a gas pycnometer (25 °C) in Table 1. The calculated detonation velocity and pressure of **2** (*D*: 8900 m s^−1^, *P*: 32.4 GPa) and **4** (*D*: 8789 m s^−1^, *P*: 29.8 GPa) approach that of RDX (8795 m s^−1^ and 34.9 GPa).

## 3. Methods and Materials

### 3.1. Safety Precaution

In this work, all compounds are potential energetic materials that tend to explode under certain external stimuli. Therefore, the whole experimental process should be carried out by using proper safety equipment, such as safety shields, eye protection and leather gloves.

### 3.2. General Methods

^1^H and ^13^C NMR spectra were tested using Bruker Avance NEO 400 MHz spectrometer (400 and 100 MHz, respectively) in *d*-DMSO. Chemical shifts are reported as δ values relative to internal standard *d*-DMSO (δ 2.50 for ^1^H NMR and 39.52 for ^13^C NMR) using Bruker TopSpin 4.0.9. Infrared spectra (IR) were obtained on a PerkinElmer Spectrum BX FT-IR instrument equipped with an ATR unit at 25 °C using an Omnic software. Elemental analyses of C/H/N were investigated on a Vario EL III Analyzer. The onset decomposition temperature was measured using a TA Instruments discovery DSC25 differential scanning calorimeter at a heating rate of 5 °C min^−1^ under dry nitrogen atmosphere. Densities were determined at room temperature by a Micromeritics AccuPyc 1345 gas pycnometer. Impact and friction sensitivities were tested by a BAM fallhammer and friction tester. X-ray diffractions of all single crystals were carried out on a Bruker D8 VENTURE diffractometer using Mo-Kα radiation (λ = 0.71073 Å). The crystal structures were produced employing Mercury 2021.1.0 software and XP. All reagents used in the experiment were purchased from Aladdin manufacturers.

### 3.3. Potassium (3-(Dinitromethanidyl)-[1,2,4]triazolo[4,3-b][1,2,4,5]tetrazin-6-yl)(nitro)amide (***1***)

Compound **S1** (1.03 g, 4.00 mmol) was added to 98% H_2_SO_4_ (20 mL) with portions at −5 °C. After being stirred for 30 min, 100% HNO_3_ (10 mL) was added dropwise below −5 °C. After addition, the sulfonitric mixture was allowed to slowly warm up to room temperature and stirred for another 10 h. The mixture was poured into ice water (300 g) under stirring and extracted with diethyl ether (3 × 30 mL). The organic phase was dried with Na_2_SO_4_ and filtered, then the methanol solution (20 mL) of KI (2.66 g, 16.00 mmol) was added dropwise. After stirring for 10 h, compound **1** was filtered and washed with methanol as a yellow solid (1.22 g, 3.37 mmol, 84.3%). *T_dec_*: 222.9 °C; ^13^C NMR (100 MHz, DMSO-d6): δ 159.3, 148.8, 141.4, 121.3; IR (cm^−1^) ν˜ = 1538, 1504, 1382, 1212, 1176, 1138, 1064, 1022, 997, 827. EA (C_4_K_2_N_10_O_6_, 361.93 amu): Calcd (%), C: 13.26; H: 0.00; N: 38.66; Found (%), C: 13.52; H: 0.32; N: 38.98. (Figures S1, S2, S9 and S13).

### 3.4. General Procedures for Synthesis of Compounds ***2***, ***3*** and ***4***

The silver salt was synthesized by reacting with AgNO_3_. To a suspension of the silver salt (2.00 mmol) in water, 3.80 mmol of corresponding hydrochloride salt was added. After stirring for 8 h at room temperature, the solid was removed by filtration. The filter cake was washed with water (100 mL) and the filtrate was removed, then dried under vacuum to give the corresponding compound.

#### 3.4.1. Ammonium (3-(Dinitromethanidyl)-[1,2,4]triazolo[4,3-b][1,2,4,5]tetrazin-6-yl)(nitro)amide (**2**)

Yellow solid (507 mg, 1.58 mmol, 83.2%). *T_dec_*: 152.1 °C; ^1^ H NMR (400 MHz, DMSO-d6): δ 7.11 (s, 8H); ^13^ C NMR (100 MHz, DMSO-d6): δ 159.3, 148.8, 141.4, 121.2; IR (cm^−1^) ν˜ = 3184, 1537, 1505, 1404, 1201, 1127, 1064, 1019, 993, 823. EA (C_4_H_8_N_12_O_6_, 320.07 amu): Calcd (%), C: 15.01; H: 2.52; N: 52.50; Found (%), C: 15.11; H: 2.12; N: 52.88. (Figures S3, S4, S10 and S14).

#### 3.4.2. Guanidine (3-(Dinitromethanidyl)-[1,2,4]triazolo[4,3-b][1,2,4,5]tetrazin-6-yl)(nitro)amide (**3**)

Yellow solid (632 mg, 1.56mmol, 82.1%). *T_dec_*: 228.4 °C; ^1^H NMR (400 MHz, DMSO-d6): δ 7.12 (s, 12H); ^13^C NMR (100 MHz, DMSO-d6): δ 159.1, 158.4, 149.0, 141.5, 121.5; IR (cm^−1^) ν˜ = 3397, 3177, 1641, 1578, 1528, 1488, 1462, 1376, 1345, 1253, 1218, 1135, 1041, 990, 827. EA (C_6_H_12_N_16_O_6_, 404.11 amu): Calcd (%), C: 17.83; H: 2.99; N: 55.44; Found (%), C: 17.66; H: 2.87; N: 55.78. (Figures S5, S6, S11 and S15).

#### 3.4.3. 3,6,7-Triamino-7*H*-[1,2,4]triazolo[4,3-b][1,2,4]triazol-2-ium(3-(dinitromethanidyl)-[1,2,4]triazolo[4,3-b][1,2,4,5]tetrazin-6-yl)(nitro)amide (**4**)

Yellow solid (900 mg, 1.51 mmol, 79.5%). *T_dec_*: 170.8 °C; ^1^H NMR (400 MHz, DMSO-d6): δ 13.29 (s, 2H), 8.20 (s, 4H), 7.23 (s, 4H), 5.71 (s, 4H); ^13^C NMR (100 MHz, DMSO-d6): δ 160.2, 158.7, 148.8, 147.5, 141.5, 141.2, 121.2; IR (cm^−1^) ν˜ = 3366, 3267, 3110, 1678, 1644, 1501, 1467, 1439, 1250, 1214, 1173, 1149, 1053, 1001. EA (C_10_H_14_N_26_O_6_, 594.16 amu): Calcd (%), C: 20.21; H: 2.37; N: 61.27; Found (%), C: 20.61; H: 2.68; N: 60.96. (Figures S7, S8, S12 and S16).

## 4. Conclusions

In conclusion, four new gem-dinitromethyl and nitroamine energetic salts with a fused 1,2,4-triazolo[4,3-b][1,2,4,5]tetrazine backbone were synthesized and their structures were characterized by IR, NMR spectroscopy and elemental analysis. The structures of compounds **3** and **4** were further confirmed by single crystal X-ray diffraction. The coordinative combination of nitroamine and gem-dinitromethyl with fused 1,2,4-triazolo[4,3-*b*][1,2,4,5]tetrazine greatly enhanced the safety properties such as impact sensitivity, friction sensitivity and thermalstability of the prepared compounds compared to those with single five-member ring or bridged heterocycles bearing the same groups. Furthermore, the oxygen balances and detonation performances were also improved compared to the precursor **S1**. The detonation performance of ammonium salt **2** with high nitrogen and oxygen content of 82.5% and density of 1.805 g cm^−3^ as well as detonation velocity of 8900 m s^−1^ are comparable to those of RDX, making it competitive as a candidate for RDX replacement. The approaches presented in this work also provide a reference for design and synthesis of energetic compounds with enhanced energy and safety.

## Figures and Tables

**Table 1:** Physicochemical and energetic properties of compounds **1**, **2**, **3** and **4**.

Compound	*T_d_^a^* (°C)	*ρ ^b^* (g cm^−3^)	Δ*H_f_^c^* (kJ mol^−1^)	*D ^d^* (m s^−1^)	*P ^e^* (Gpa)	*IS ^f^* (J)	*FS ^g^* (N)	N + O *^h^* (%)
**1**	222.9	2.062	172.6	8040	28.1	15	160	65.2
**2**	152.2	1.805	439.7	8900	32.4	15	240	82.5
**3**	228.4	1.629	485.1	7892	23	40	360	79.2
**4**	170.8	1.817	1627.3	8789	29.8	40	160	77.4
**S1**	197	1.756	564.3	8574	29.4	35	240	79.1
RDX	204	1.8	70.3	8795	34.9	7.5	120	81.1

*^a^* Decomposition temperature from DSC (5 °C min^−1^). *^b^* Density measured using a gas pycnometer at 25 °C. *^c^* Heat of formation—Gaussian 09 (Revision E.01). *^d^* Detonation velocity—EXPLO5 V6.05. *^e^* Detonation pressure—EXPLO5 V6.05. *^f^* Impact sensitivity. *^g^* Friction sensitivity. *^h^* Nitrogen-oxygen content.

## Data Availability

Not applicable.

## Supplementary Materials

The following supporting information can be downloaded at: https://www.mdpi.com/article/10.3390/ijms232214337/s1. References [21,22] are cited in the Supplementary Materials.
