# Supplementary material for: Coordinative Combination of Nitroamine and Gem-Dinitromethyl with Fused Ring for Enhanced Oxygen Balances and Detonation Properties
Source: Int J Mol Sci. 2022 Nov 18;23(22):14337. doi: 10.3390/ijms232214337 (PMC9697839; doi:10.3390/ijms232214337)
Supplement: Supplementary file 1 [file ijms-23-14337-s001.zip › ijms-2018143-supplementary.pdf]

Supplementary Materials

Coordinative Combination of Nitroamine and Gem-Dinitromethyl with Fused Ring for Enhanced Oxygen Balances and Detonation Properties

Xun Zhang 1,2, Yaxi Wang 1,2, Xinyuan Zhao 1,2, Chunlin He 1,2,3,4,* and Siping Pang 1,*
[1]*School of Materials Science & Engineering, Beijing Institute of Technology, Beijing 100081, China*
[2]*Experimental Center of Advanced Materials, School of Materials Science & Engineering, Beijing Institute of Technology, Beijing 100081, China*
[3]*Yangtze Delta Region Academy, Beijing Institute of Technology, Jiaxing 314019, China.*
[4]*Chongqing Innovation Center, Beijing Institute of Technology, Chongqing 401120, China.*
* Correspondence: chunlinhe@bit.edu.cn (C.H.); pangsp@bit.edu.cn (S.P.)

Contents

1. ^{1}H and ^{13}C NMR spectra S2
2. IR spectra S6
3. DSC curves of compound 1, 2, 3, 4 S7
4. X-ray Crystallography S9
5. Heats of formation S10
6. References S12

1. ^{1}H and ^{13}C NMR spectra

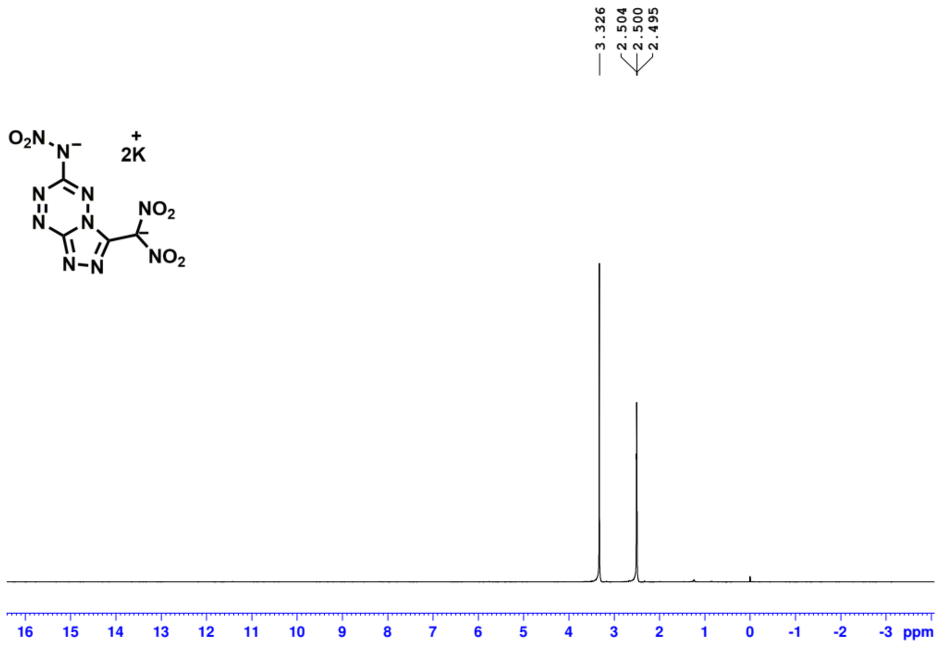

Figure S1. ^{1}H NMR spectrum of **1** in d_6-DMSO.

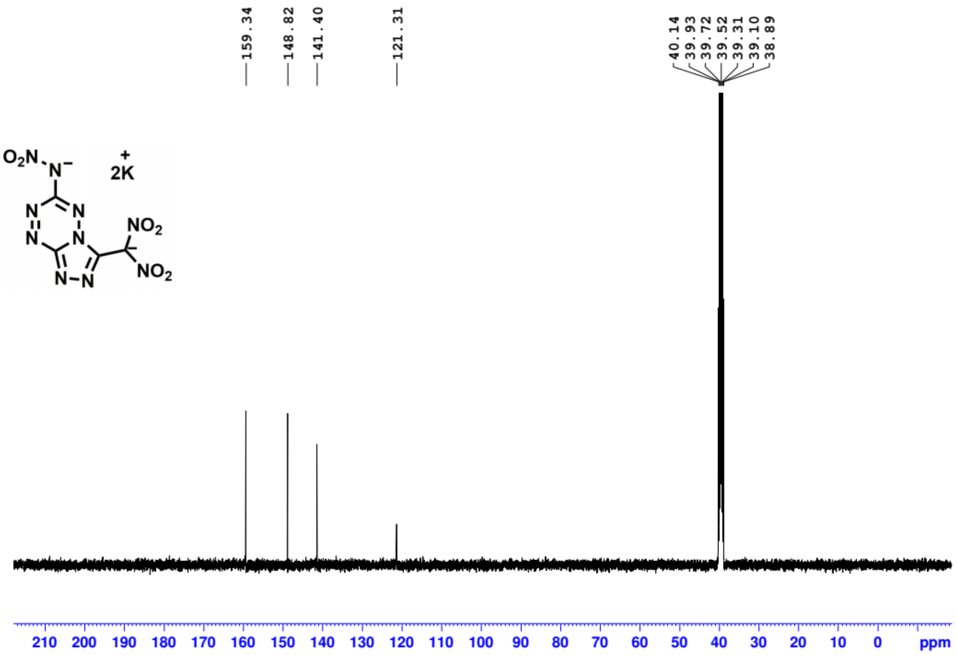

Figure S2. ^{13}C NMR spectrum of **1** in d_6-DMSO.

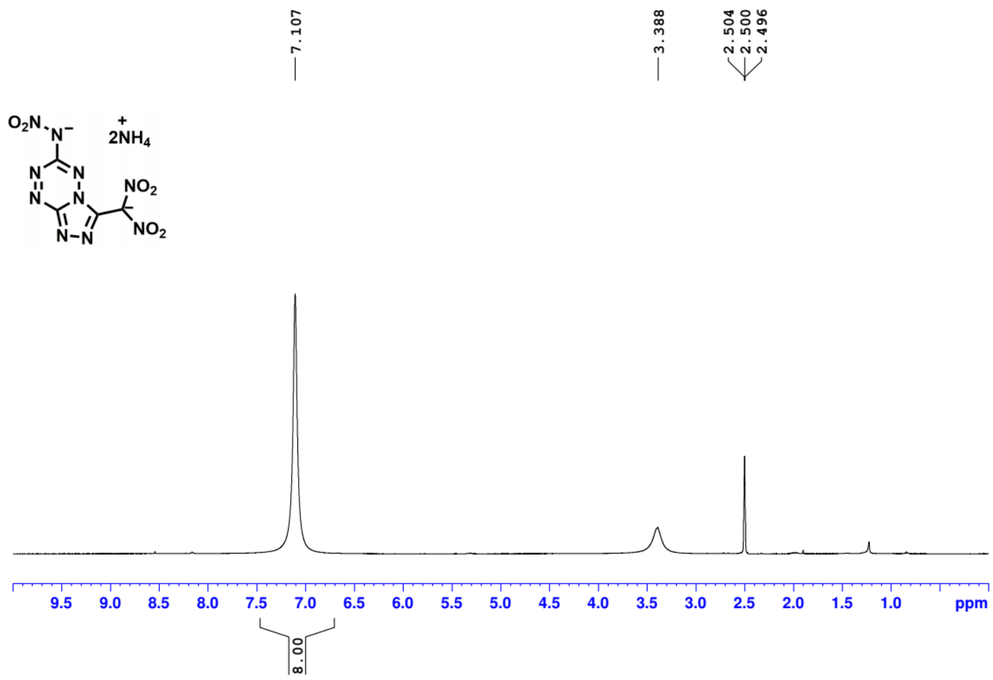

Figure S3. ^{1}H NMR spectrum of **2** in d_6-DMSO.

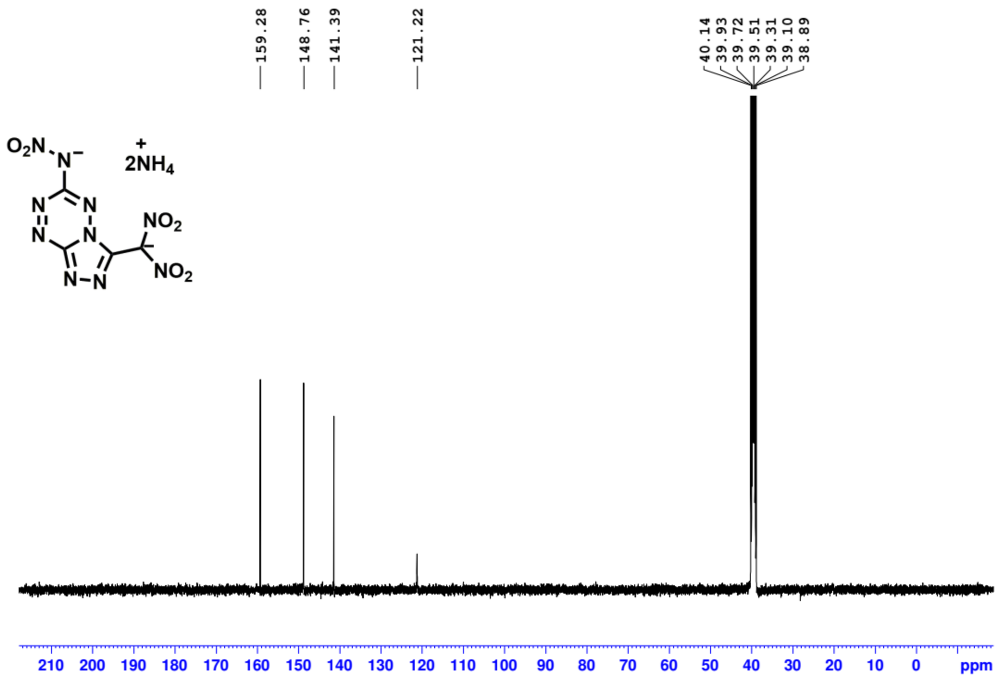

Figure S4. ^{13}C NMR spectrum of **2** in d_6-DMSO.

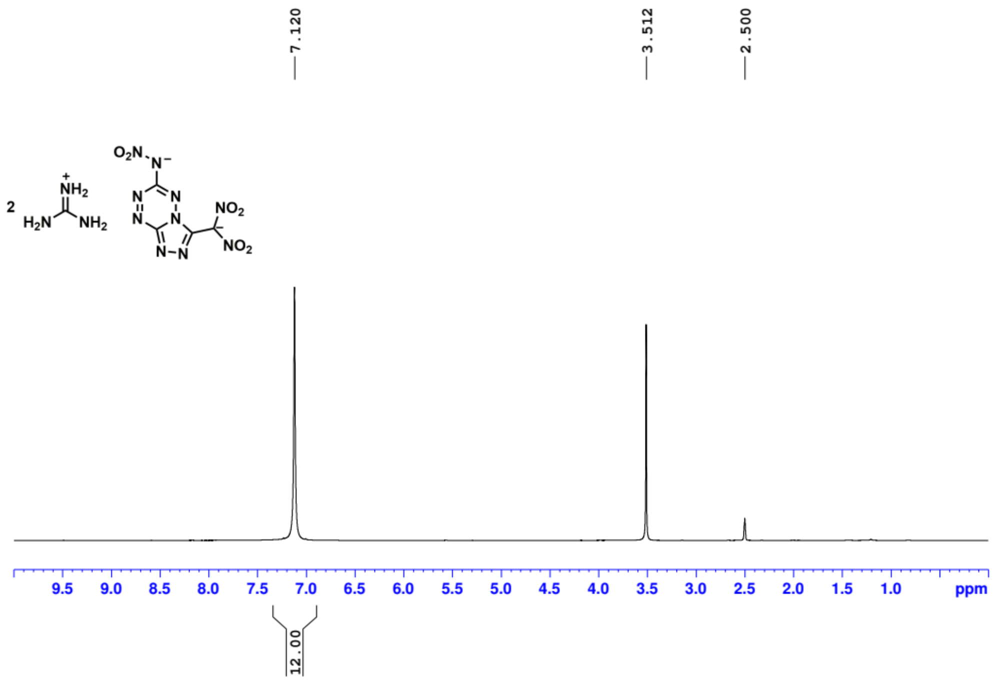

Figure S5. ^{1}H NMR spectrum of **3** in d_6-DMSO.

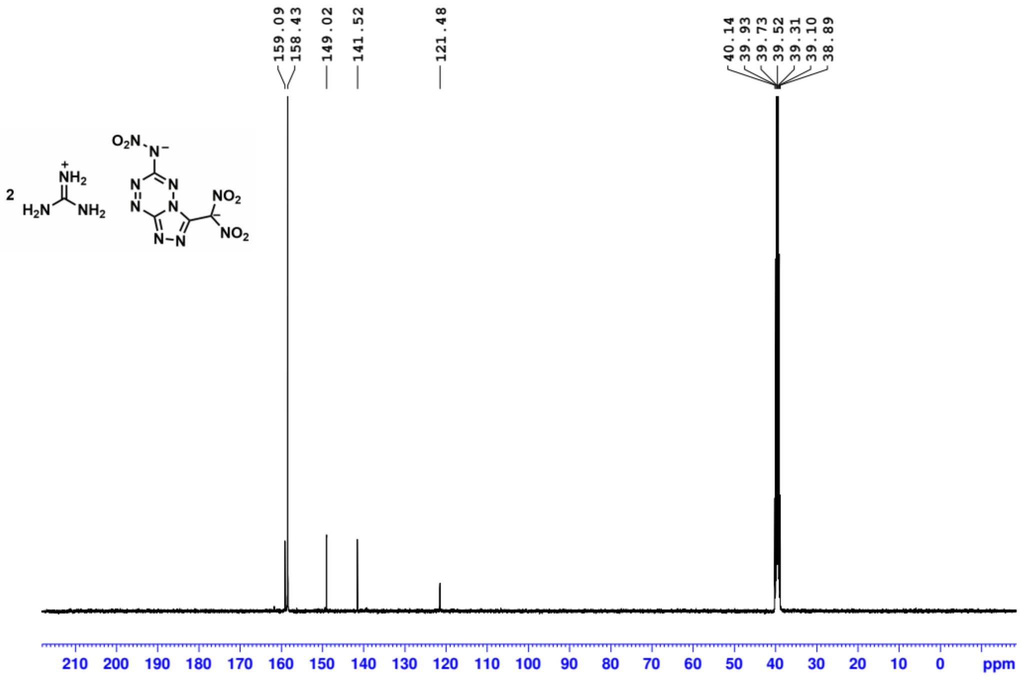

Figure S6. ^{13}C NMR spectrum of **3** in d_6-DMSO.

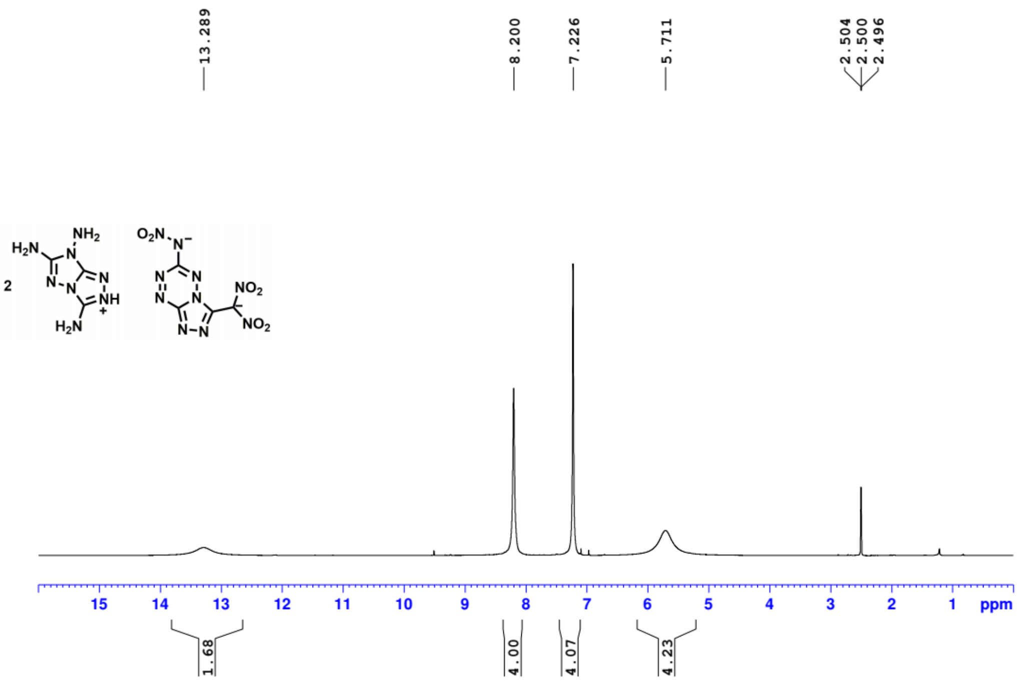

Figure S7. ^{1}H NMR spectrum of **4** in d_6-DMSO.

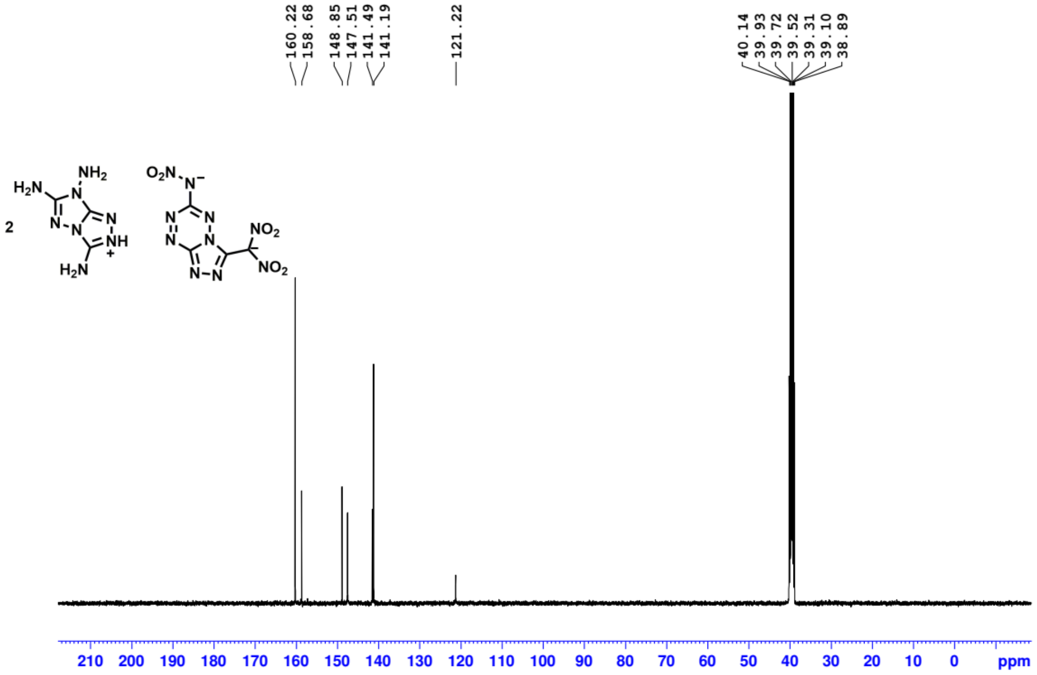

Figure S8. ^{13}C NMR spectrum of **4** in d_6-DMSO.

2. IR spectra

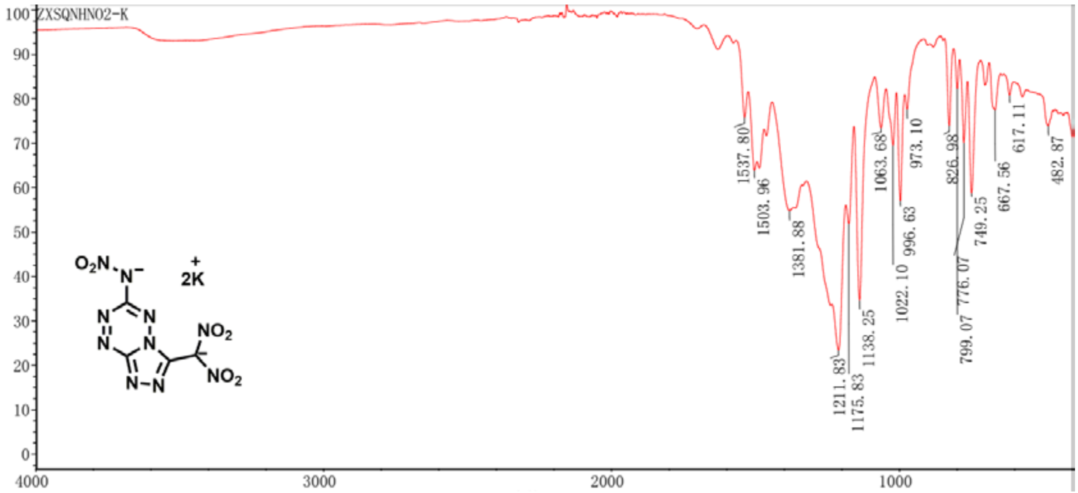

Figure S9. IR spectrum of compound **1**

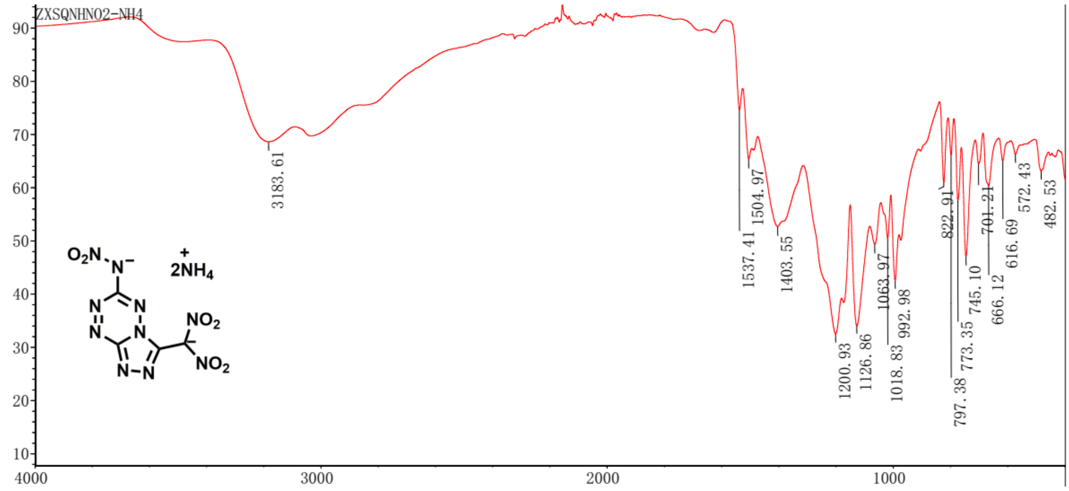

Figure S10. IR spectrum of compound **2**

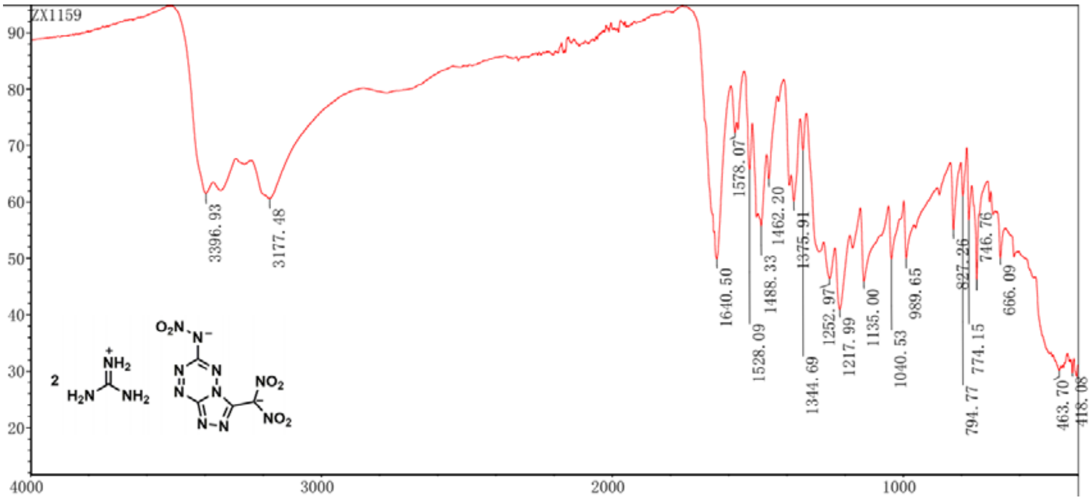

Figure S11. IR spectrum of compound **3**

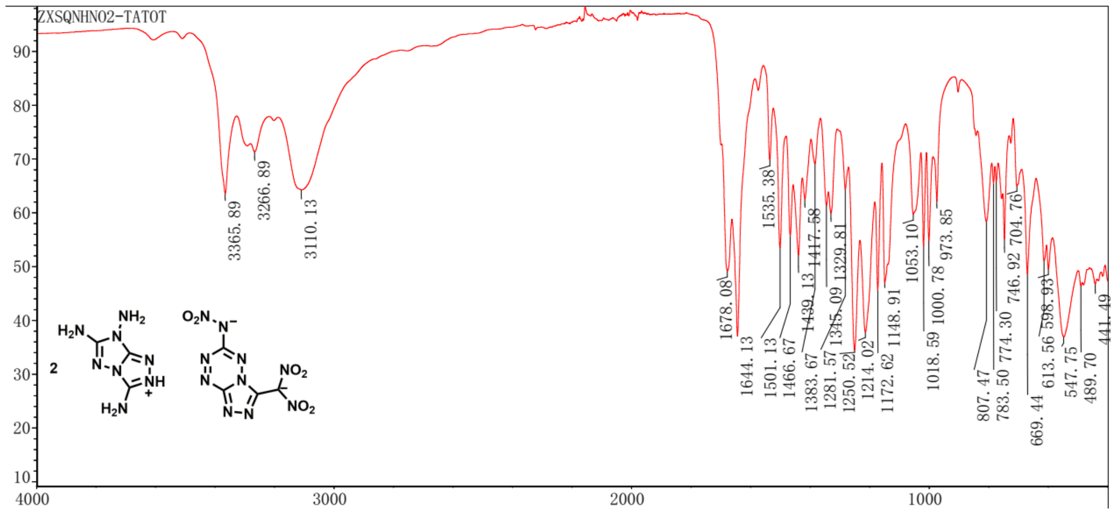

Figure S12. IR spectrum of compound **4**

3. DSC curves of compound 1, 2, 3

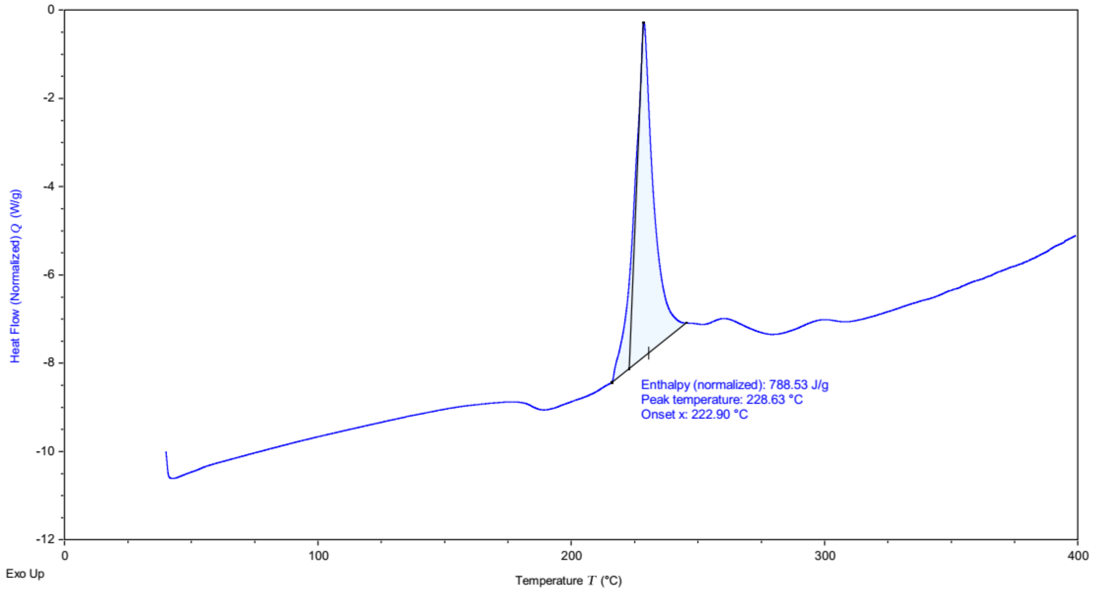

Figure S13. DSC curve of compound **1** at 5 °C min^{-1}

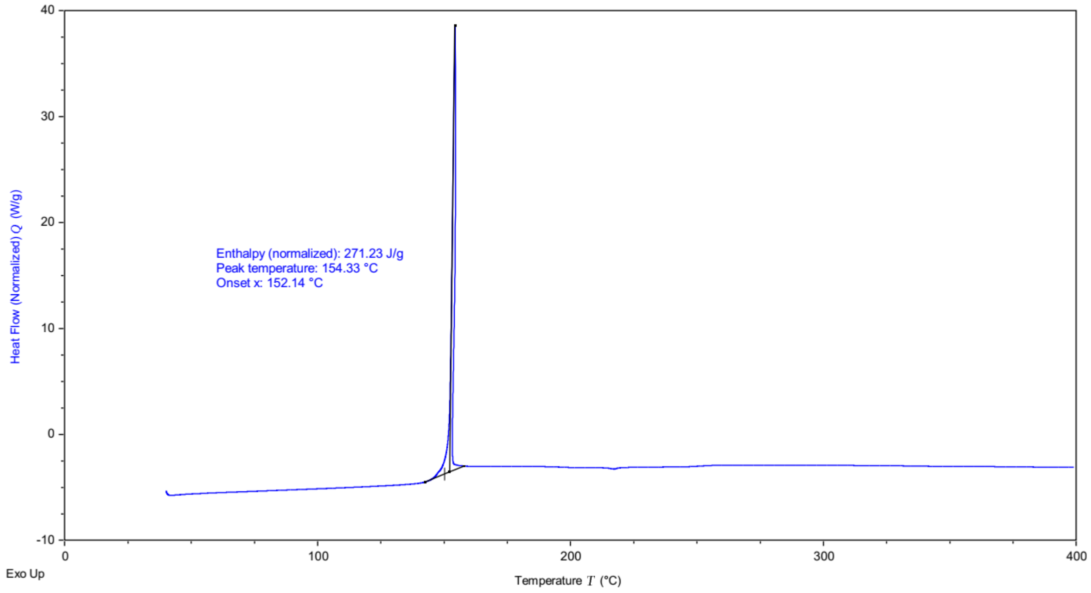

Figure S14. DSC curve of compound **2** at 5 °C min^{-1}

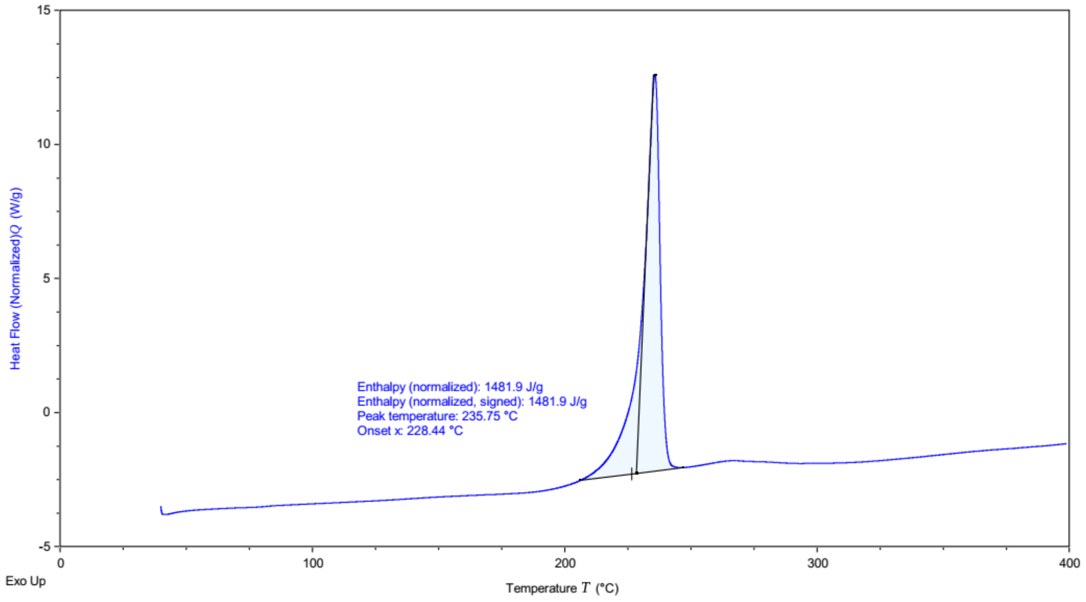

Figure S15. DSC curve of compound **3** at 5 °C min^{-1}

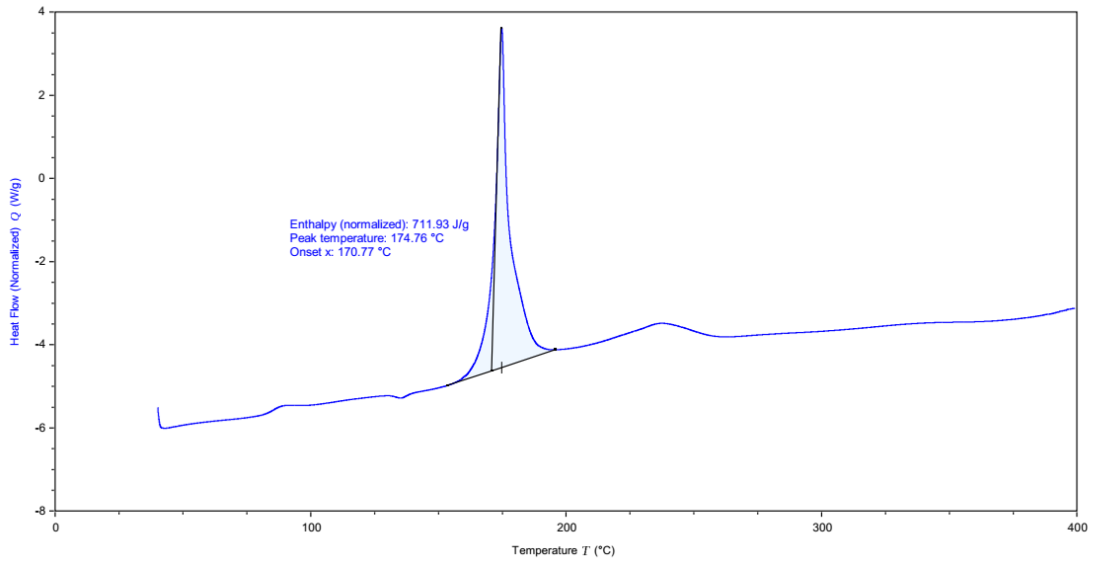

Figure S16. DSC curve of compound **4** at 5 °C min^{-1}

4. X-ray Crystallography

Table S1 The single crystal diffraction refinement details for compound **3** and **4**

**Compound**	3	4
CCDC No.	2204894	2204893
Empirical formula	C6H12N16O6	C10H16N26O7
Formula weight	404.32	612.49
Temperature/K	296(2)	298
Crystal system	monoclinic	monoclinic
Space group	Cc	P21/c
a/Å	10.09(4)	23.205(4)
b/Å	24.07(9)	11.3686(16)
c/Å	7.28(3)	8.9053(14)
α/°	90	90
β/°	111.16(4)	100.275(5)
γ/°	90	90
Volume/Å3	1650(10)	2311.6(6)
Z	4	4
ρcalcg/cm3	1.628	1.76
μ/mm-1	0.142	0.149
F(000)	832	1256
Crystal size/mm3	0.16 × 0.14 × 0.05	0.11 × 0.04 × 0.02
Radiation	MoKα (λ = 0.71073)	MoKα (λ = 0.71073)
2θ range for data	4.648 to 55.81	4.002 to 50.798

collection/°		
Index ranges	-12 ⩽ h ⩽ 12, -31 ⩽ k ⩽ 30, -9 ⩽ l ⩽ 9	-27 ⩽ h ⩽ 27, -12 ⩽ k ⩽ 13, -10 ⩽ l ⩽ 10
Reflections collected	7000	15668
Independent reflections	3574 [Rint = 0.1192, Rsigma = 0.2075]	4225 [Rint = 0.1108, Rsigma = 0.1069]
Data/restraints/parameters	3574/3/253	4225/8/406
Goodness-of-fit on F2	0.984	1.032
Final R indexes [I>=2σ (I)]	R1 = 0.0670, wR2 = 0.1321	R1 = 0.0745, wR2 = 0.1595
Final R indexes [all data]	R1 = 0.1712, wR2 = 0.1616	R1 = 0.1753, wR2 = 0.2119
Largest diff. peak/hole / e Å-3	0.39/-0.41	0.47/-0.46

5. Heats of formation

Theoretical calculations were performed by using the Gaussian 09 suite of programs [1,2]. Gas phase heats of formation of the three energetic salts were computed based on an isodesmic reaction (Scheme. S6). The geometric optimization and frequency analyses of the structures were calculated by B3LYP/6-31+G** level. The gas phase enthalpy of formation was calculated and the enthalpy of reaction was gained by combining the MP2/6-311++G** energy difference for the reactions, the scaled zero point energies (ZPE), values of thermal correction (HT), and other thermal factors.

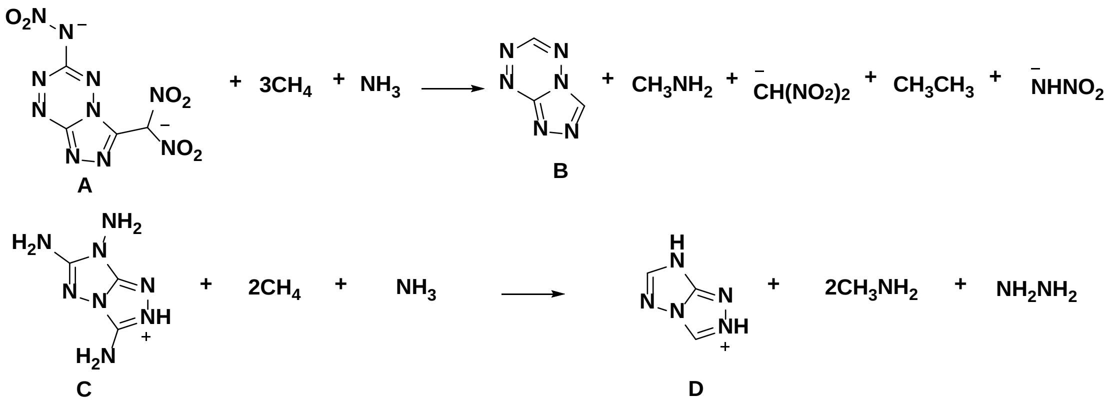

Scheme S1. Isodesmic reactions.

The XYZ coordinates for the optimized structure for the anion of compounds **1-4**

Atom	X	Y	Z
N	-2.57278000	1.19927600	-0.24747300
N	-1.79407800	2.21314900	-0.09912200
C	-0.46148300	1.97951200	0.02184800
N	0.03390400	0.69538600	-0.04668000
N	-0.74156500	-0.40806300	-0.17429600
C	-2.03088500	-0.11324900	-0.27860100
N	0.53807800	2.85118400	0.16753000
N	1.67980200	2.11999800	0.19367300
C	1.38943900	0.81624900	0.06296500
N	-2.87906600	-1.13492200	-0.60400400
C	2.34974500	-0.27816700	0.03348000
N	2.86336100	-0.66198300	-1.20525300
O	3.73939800	-1.54097500	-1.32634600
O	2.38205100	-0.06068500	-2.20640900
N	2.74203500	-0.83990800	1.24876100
O	3.62432400	-1.71749600	1.32876100
O	2.14598400	-0.40070400	2.27127500
N	-4.04870100	-1.13849800	0.04331000
O	-4.20584500	-0.52157800	1.13035500
O	-4.96439100	-1.84548700	-0.44429500

The XYZ coordinates for the optimized structure for TATOT cation

Atom	X	Y	Z
C	1.45246200	-0.83788700	0.00079100
N	0.29368600	-1.48444300	0.00086800
N	-0.61038000	-0.42841100	-0.00056800
C	-0.03325700	0.81352200	-0.00030000
N	1.31662900	0.56167600	0.00060500
C	-1.94523200	-0.25496300	0.00082300
N	-2.09782800	1.08889600	0.00255100
N	-0.89040700	1.79504100	0.00155900
N	2.40319800	1.42766100	-0.00184800
N	2.66676300	-1.38757800	0.00224400
N	-2.86614800	-1.22455500	-0.01570000
H	-2.96191500	1.61496200	-0.00546300
H	2.38754500	2.01046700	-0.83752000
H	2.39021800	2.01175200	0.83295700
H	3.48303300	-0.78922000	-0.00272000
H	2.77281500	-2.39233700	-0.00359000
H	-3.85454100	-1.02583500	0.05218200
H	-2.56958200	-2.19182800	0.02828300

6. References

[1] Becke, A.D. Density-functional thermochemistry. III. The role of exact exchange. *J. Phys. Chem.* **1993***, 98,* 5648.

[2] Stephens, P.J.; Devlin, F.J.; Chabalowski, C.F.; Frisch, M.J. Ab Initio Calculation of Vibrational Absorption and Cir-cular Dichroism Spectra Using Density Functional Force Fields J. Phys. Chem. 1994, 98, 11623.
